# Integrating carbon stocks and landscape connectivity for nature‐based climate solutions

**DOI:** 10.1002/ece3.9725

**Published:** 2023-01-09

**Authors:** Paul O'Brien, John S. Gunn, Alison Clark, Jenny Gleeson, Richard Pither, Jeff Bowman

**Affiliations:** ^1^ Ontario Ministry of Natural Resources and Forestry Ontario Peterborough Canada; ^2^ New Hampshire Agricultural Experiment Station University of New Hampshire Durham New Hampshire USA; ^3^ Environment and Climate Change Canada Ottawa Ontario Canada

**Keywords:** biodiversity protection, climate change mitigation, conservation planning, corridors and connectivity, nature‐based solutions, protected areas

## Abstract

Actions to protect against biodiversity loss and climate change will require a framework that addresses synergies between these interrelated issues. In this study, we present methods for identifying areas important for the implementation of nature‐based climate solutions and biodiversity conservation by intersecting high‐resolution spatial data for carbon storage and landscape connectivity. We explored the spatial congruence of carbon and connectivity in Ontario, Canada and examined effectiveness of current protected areas coverage. We found a weak positive relationship between carbon stocks and landscape connectivity; however, our maps revealed large hotspots, with high values of both indices, throughout the boreal forest and northern peatlands and smaller, isolated hotspots, in the settled landscapes of the south. Location of hotspots varied depending on whether we considered forest or soil carbon. Further, our results show that current protected and conserved areas in Ontario only cover 13% of landscapes with the highest values for both carbon storage and connectivity. Protection or restoration of areas that maximize the co‐benefits of carbon storage and connectivity would make significant contributions toward ambitious national targets to reduce greenhouse gas emissions and conserve biodiversity.

## Graphical abstract

We identified areas important for the implementation of nature‐based climate solutions and biodiversity conservation in Ontario, Canada by intersecting high‐resolution spatial data for carbon storage and landscape connectivity. We found large hotspots, with high values for both indices, throughout the boreal forest and northern peatlands of the province. Further, our results show that current protected and conserved areas in Ontario only cover 13% of landscapes with the highest values for both carbon storage and connectivity suggesting these areas are priorities for protection and would make significant contributions toward ambitious national targets to reduce greenhouse gas emissions and conserve biodiversity.

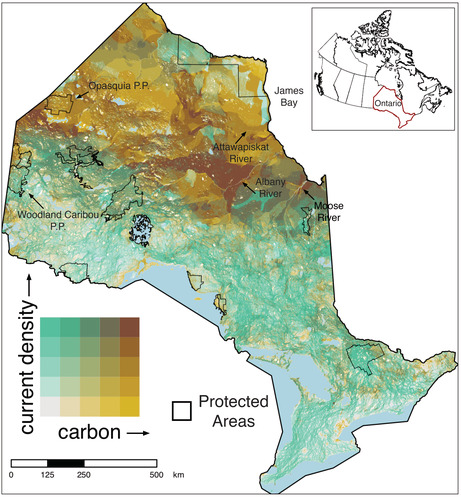

## 1 INTRODUCTION

Trends in global climate change and biodiversity loss continue to be two of the most pressing threats facing humans and nature. Avoiding catastrophic outcomes requires immediate large scale, coordinated international, national, and subnational efforts. While strategies to address both global crises have traditionally been treated separately (e.g., United Nations' Framework Convention on Climate Change versus Convention on Biological Diversity; Arneth et al., 2020; Shin et al., 2022), interdependencies between the two suggest that these issues should be addressed holistically and synergistically (Seddon et al., 2021; Turney et al., 2020). Nature‐based solutions have emerged as a promising framework to meet both climate change and biodiversity goals.

Nature‐based solutions (NbS), as defined by the International Union for Conservation of Nature (IUCN), are actions to protect, manage, and restore natural ecosystems with the aims of addressing societal issues (e.g., mitigating climate change), while simultaneously providing benefits to human wellbeing and biodiversity (Cohen‐Shacham et al., 2016). NbS are borne from the knowledge that sustainable ecosystem management, ecological restoration, and protected and conserved areas can reduce carbon emissions and increase carbon stored in plants and soil. The term NbS has been used as an umbrella for a range of actions including ecosystem‐based adaptation, ecosystem restoration, natural climate solutions, green/blue infrastructure, and protected areas (Cohen‐Shacham et al., 2019; Nesshöver et al., 2017; Seddon et al., 2020). Many government and non‐government NbS efforts have focused on restorative actions, such as planting trees (Seddon et al., 2021). Ecosystem restoration is an important part of the solution; however, it should not be seen as a silver‐bullet (Holl & Brancalion, 2020). While restoration efforts (e.g., reforestation of mixed, native forests) provide meaningful biodiversity benefits (Wang et al., 2021) and increase carbon sequestration and storage (Lewis et al., 2019), proactively conserving large intact ecosystems with high ecological integrity should remain a focus (Cook‐Patton et al., 2021; Grantham et al., 2020; Locke et al., 2019; Noon et al., 2021). Protection of intact ecosystems can be more cost effective than restoration of degraded habitats (Cook‐Patton et al., 2021; Drever et al., 2021; Watson et al., 2018) and provide multiple, synergistic benefits by maintaining existing carbon sinks and preventing large, potentially irrecoverable carbon emissions while providing biodiversity benefits (Arneth et al., 2020). Given the multitude of benefits provided by intact landscapes in their current state, protection of these areas can enable national and subnational strategies to more immediately maximize synergies between climate change mitigation and biodiversity goals, while also conserving other essential ecosystem services.

Canada is a signatory to both the United Nations Framework Convention on Climate Change (UNFCCC) and the Convention on Biological Diversity (CBD). In accordance with these international agreements, parties committed to keep global warming to within 1.5–2°C (Paris Agreement) and protect at least 17% of lands and 10% of inland waters by 2020 (CBD; Aichi Target 11). Canada has declared it will reduce greenhouse gas emissions by 40% by 2030 and reach net‐zero emissions by 2050 through cuts in emissions, innovation and protection and restoration of lands and biodiversity (Government of Canada, 2022). Moreover, Canada has committed to protecting 25% of its land area by 2025 and 30% by 2030 (ECCC, 2020; Secretariat of the CBD, 2020). Despite having some of the largest remaining intact forests (Watson et al., 2018; Wells et al., 2020) and peatlands (Goldstein et al., 2020; Noon et al., 2021), both of which provide globally significant carbon storage and safeguarding for biodiversity (Harris et al., 2021; Kurz et al., 2013; Stralberg, Arseneault, et al., 2020; Wells et al., 2020), only 13.5% of terrestrial lands in Canada are currently protected (ECCC, 2021). Given Canada's climate and biodiversity commitments and the urgency for action, there is great benefit to identifying areas that maximize co‐benefits of climate change mitigation and biodiversity protection when planning for new protected and conserved areas as well as prioritizing ecosystem restoration.

One of the challenges of expanding protected and conserved areas coverage is determining which areas are most critical to protect (Carroll & Ray, 2021). With respect to climate change mitigation, this can be as straightforward as locating and subsequently protecting areas that store and sequester, or have the potential to sequester, a relatively large amount of carbon through restoration or changes to land management approaches (Carroll & Ray, 2021; Goldstein et al., 2020). It is important to note that areas storing large amounts of carbon may not overlap with areas actively sequestering large amounts of new carbon from the atmosphere. Identifying areas important for biodiversity is a more complicated undertaking given the many definitions and methods to measure biodiversity as well as the paucity of data in some regions and for particular species (Busch & Grantham, 2013; Soto‐Navarro et al., 2020).

A commonly employed method to identify biodiversity hotspots has been to assess species richness or other species‐based metrics (Marchese, 2015; Soto‐Navarro et al., 2020). Key Biodiversity Areas (KBAs) is an emerging approach, guided by global and national standards, for identifying sites that are especially important for the conservation of biodiversity (Smith et al., 2019). However, protection of these biodiversity hotspots alone is not enough, and it is recognized that the design of well‐connected networks of protected and conserved areas is integral to global and national biodiversity efforts. Landscape connectivity is critical for species movement and gene flow through dispersal and migratory movements (Noss et al., 2012) and reductions in connectivity have been found to be a strong driver of species extinctions (Hooftman et al., 2016; Thompson et al., 2017). Maintaining or restoring landscape connectivity is cited as one of the most important biodiversity adaptation strategies, particularly in the face of climate change as species ranges shift to track suitable climates (Heller & Zavaleta, 2009; Schloss et al., 2022).

Despite connectivity being essential for the long‐term persistence of biodiversity (Ward et al., 2020) and recent advances in connectivity mapping (Dickson et al., 2019; Hall et al., 2021; Phillips et al., 2021), it is rarely incorporated into conservation planning (Carroll & Ray, 2021; Maxwell et al., 2020). Thus, we suggest that landscape connectivity should be better incorporated into planning for protected and conserved areas and priority restoration sites and integrated with areas important for carbon storage as well as areas which could be restored to enhance carbon sequestration. Here, we focus on the former (i.e., carbon storage). Together, carbon and connectivity attributes can provide a framework to consider synergies between climate change mitigation and biodiversity conservation.

Although there is an increasing recognition of the value of NbS in achieving climate change and biodiversity loss targets (IPBES, 2019; IPCC, 2022a, 2022b), few studies exist to aid national and regional efforts in identifying areas suitable for the implementation of nature‐based solutions (Zhu et al., 2021). Recent mapping efforts have identified hotspots for multiple ecosystem services across Canada, including carbon storage, freshwater, and recreational capacity (Mitchell et al., 2021). Although a KBA Canada Coalition is in the process of applying the global standards in Canada for terrestrial and freshwater areas, the results are not yet ready (http://www.kbacanada.org). Soto‐Navarro et al. (2020) have produced maps highlighting global hotspots for biodiversity and carbon storage. Similarly, Dinerstein et al. (2020) identified globally important areas for protection of biodiversity and climate stabilization, while also incorporating an analysis of connectivity among existing protected areas. These studies are excellent for mapping broad‐scale patterns of biodiversity and climate change stabilization. In Canada, however, the vast majority of public land is administered by the provinces, territories, and Indigenous governments (Carroll & Ray, 2021). Consequently, the creation of new protected and conserved areas and priority restoration sites requires coordination with provincial/territorial, Indigenous governments, local organizations, and regional land‐use planners and will require more fine‐scale resolution mapping resources.

Sothe et al. (2022) have produced a map of terrestrial carbon stocks (above and below‐ground) in Canada at a resolution useful for regional, Indigenous, and local governments. These maps indicate that Canada possesses large stores of terrestrial carbon, much of which is considered irrecoverable and if lost would significantly contribute to atmospheric carbon levels (Noon et al., 2021). Recent advances have also been made with respect to connectivity mapping in Canada. Pither et al. (2021) have used circuit theory (McRae & Beier, 2007) to produce the first pan‐Canadian current density map, which reflects the probability of an animal moving through any point in a landscape and can be used to identify areas important for connectivity. This new connectivity map is also at a resolution useful for both national and regional land‐use planners and has been validated using independent wildlife data. These recent advances in high‐resolution mapping resources for carbon storage and landscape connectivity provide the opportunity for the first time to explore the spatial relationship between these two important values.

Here, we focus on Ontario, the second largest province in Canada, as a case study of how regional climate and biodiversity actions can support national targets. Ontario stands out as an area of interest because of the juxtaposition of large, intact natural areas and those significantly affected by human modification (Hirsh‐Pearson et al., 2022) and because of its large carbon stocks (Sothe et al., 2022). Using high‐resolution spatial data, we employ methods to examine the intersection of existing carbon storage and connectivity maps to identify important areas for conservation, including those that may be a priority for future restoration. We also explore the relationship of these two metrics by testing the hypothesis that since sites with high carbon stocks also tend to have low cost for animal movement (i.e., they generally occur in areas with a low human footprint; Barnett & Belote, 2021), these high carbon sites should also contribute disproportionately to connectivity. We predicted a positive relationship between movement probability estimates from circuit theory and carbon stocks. We further predicted that areas most important for carbon storage and connectivity will be large intact landscapes.

## 2 METHODS

We used a bivariate mapping approach to explore the spatial congruence of carbon and connectivity layers in Ontario and examine how effective current protected areas coverage is at conserving hotspots between these layers. We then use the resulting maps to make recommendations for areas in Ontario that would be most effective at maximizing synergies between carbon storage and connectivity and thus should be considered high priorities for protection.

### 2.1 Carbon data

As a measure of climate change mitigation value, we used a recently created 250‐m resolution map of terrestrial carbon stocks of Canada (Sothe et al., 2022). The researchers produced separate maps of existing forest carbon and soil carbon stocks. The forest carbon layer includes estimates of above‐ground live biomass, dead plant matter, and below‐ground root biomass and the soil organic carbon layer provides estimates of carbon stocks for the top 1‐m depth across Canada. Since the estimates of carbon contained within forests is comprised of both above‐ground (live and dead plant matter) and below‐ground (root biomass) biomass, we adopt the terms forest and soil carbon to distinguish between these two different carbon pools. Both the forest and soil carbon stock maps were produced using a combination of field measurements (including peatland samples), satellite data, climate and topographic variables, and a machine learning algorithm, which provides significant advances to estimates of terrestrial carbon stocks in Canada at such a high resolution. For the present study, we first cropped raster extents to that of Ontario and then resampled layers to a spatial resolution of 300‐m to match that of the connectivity map. Due to the large differential in carbon stock sizes between the forest and soil carbon pools, we explored the relationship of each layer with connectivity separately. Sothe et al. (2022) reported that Canadian forests store approximately 18.4 Pg C, compared with the 306 Pg C of soil organic carbon (SOC) stored in the top 1‐m. Given that carbon stocks contained within the soil carbon pool in Canada are vastly larger than that of the forest carbon pool, we decided to separate these two layers for the following analysis.

### 2.2 Connectivity layer

To identify areas important for connectivity, we used the 300‐m resolution current density map of Canada produced by Pither et al. (2021). The authors modeled connectivity across Canada using a circuit theory approach, which draws on similarities between animal movement through a landscape and flow of electricity through a circuit, allowing for identification of multiple movement pathways. Electricity travels through a circuit according to a random walk, and this property allows animal movement as a random walk (Doyle & Snell, 1984) to be modeled using principles from electric circuits. Circuit theory models require a movement cost surface as an input, which represents anthropogenic and natural landscape features by the degree to which they impede or facilitate movement of animals. The cost surface used by Pither et al. (2021) was produced using the most up to date spatial data including the Canadian human footprint (Hirsh‐Pearson et al., 2022) and a recently developed national road layer (Poley et al., 2022). In their study, the authors modeled terrestrial connectivity (i.e., landscape connectivity relevant to terrestrial, non‐volant fauna), such that natural features like forests and wetlands represented a low cost to movement, while roads and cities as well as large water bodies and mountains represented a high cost to movement (for more details see Pither et al., 2021). This approach models landscape connectivity based on the degree of “naturalness” (the inverse of human modification) or ecological integrity under the assumption that more natural landscapes are less costly and therefore better facilitate animal movement and flow of ecological processes (Krosby et al., 2015; Spencer et al., 2010; Theobald et al., 2012). The resulting current density map output reflects the probability of movement of an animal through any given point across the landscape. Current density represents the amount of electrical current (measured in amps (A)) flowing through a given cell and is analogous to the probability of a random walker using that location. Current density in a 300‐m pixel is proportional to the probability of that pixel being used during a random walk through the province. The authors used a wall‐to‐wall method of Circuitscape implemented in Julia (Hall et al., 2021; Phillips et al., 2021) to produce the first pan‐Canadian, multi‐species current density map at a resolution fine enough to support regional actions, such as for our provincial scale study.

### 2.3 Comparing carbon and connectivity

To test the relationship between carbon storage and connectivity, we evaluated Spearman rank correlations between current density values and raster values of both forest and soil carbon. We used a bivariate mapping approach to identify important areas of overlap between current density and carbon layers. We calculated quantiles at 20% intervals for the current density, forest, and soil carbon layers and then reclassified cell values for each raster layer with a value of 1–5 based on which percentile range they fell within. We then overlapped the current density map with each carbon map to produce two bivariate maps for current density by forest carbon and current density by soil carbon. Cell values for the bivariate maps were calculated using all unique combinations of the two layers, which resulted in 25 different cell values (5 × 5 matrix; e.g., Soto‐Navarro et al., 2020) varying in degree of importance for each layer. We followed a similar approach to Mitchell et al. (2021) and Soto‐Navarro et al. (2020) such that areas where cells from both layers are within the top 20% represent areas most important for protection, while areas in the lowest 20% for both layers may indicate areas important for restoration. We use the phrase “hotspot” to refer to areas with the highest quantiles (top 20%) for both connectivity and carbon overlapping.

### 2.4 Overlap of existing protected areas with carbon and connectivity hotspots

To examine how existing protected areas overlap with hotspots for carbon storage and connectivity, we calculated the proportion of each quantile combination within protected areas boundaries. First, we calculated the area covered by each of the 25 quantile combinations. Then, using a vector layer of Ontario protected and conserved areas from the Canadian Protected and Conserved Areas Database (ECCC, 2021), we extracted raster values from each of the maps and calculated percent area protected within each group. Using anticipated targets for terrestrial land conservation based on draft content for the Post‐2020 Global Biodiversity Framework (Secretariat of the CBD, 2020), we identified protection of 30% to be a goal for protection.

## 3 RESULTS

**FIGURE 1:**
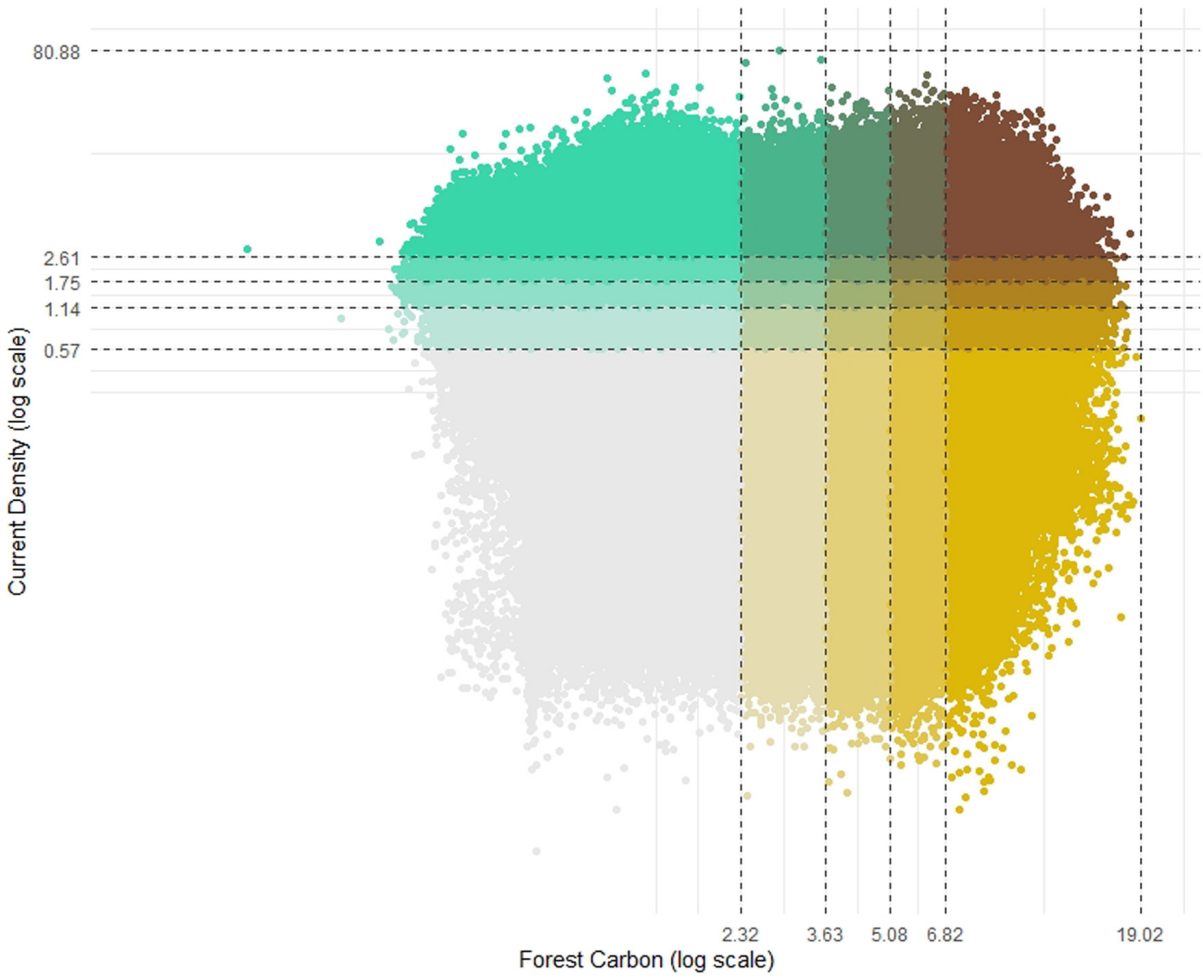
Scatterplot of cell values from forest carbon and current density, representing the probability of animal movement, raster layers for Ontario. Values are divided into quantile groups (by 20% intervals) with the color gradient expressing overlap between percentile groups for both datasets. Points in the top right corner (dark brown) represent areas where cell values for both layers are in the top 20% quantile representing high connectivity and high carbon storage.

**FIGURE 2:**
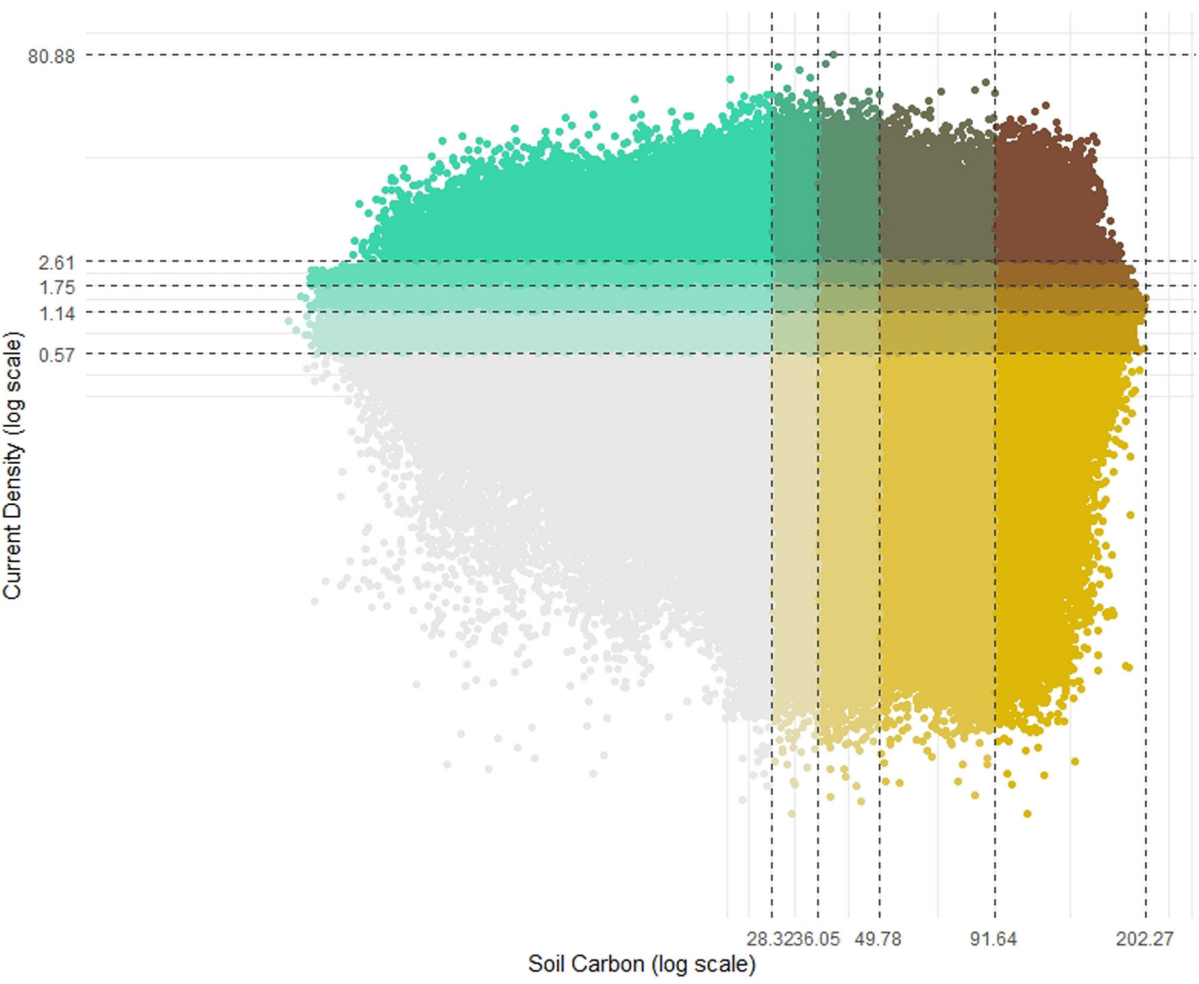
Scatterplot of cell values from soil carbon and current density, representing the probability of animal movement, raster layers for Ontario. Values are divided into quantile groups (by 20% intervals) with the color gradient expressing overlap between percentile groups for both datasets. Points in the top right corner (dark brown) represent areas where cell values for both layers are in the top 20% quantile representing high connectivity and high carbon storage.

**FIGURE 3:**
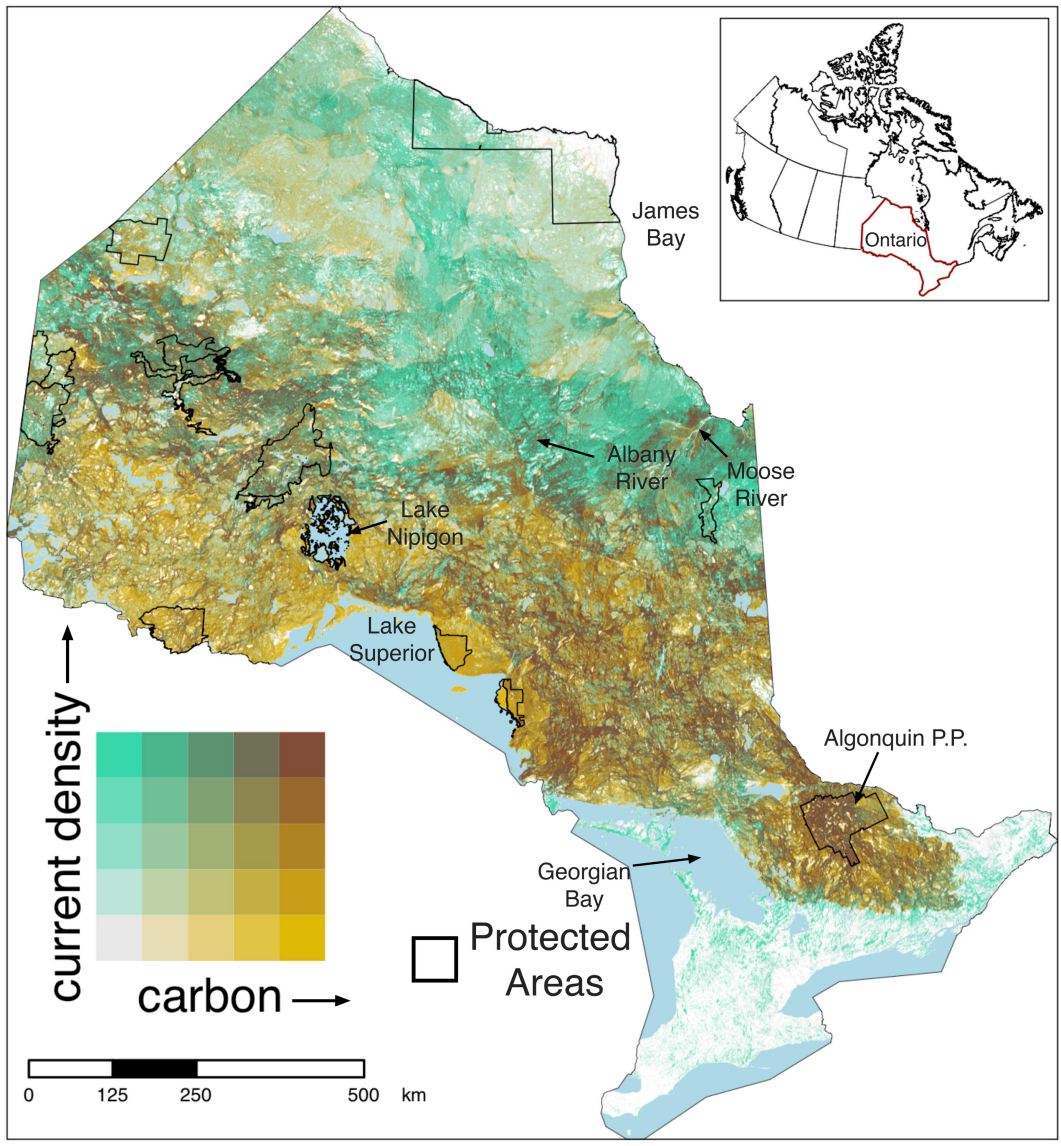
Bivariate map showing spatial overlap between current density, representing the probability of terrestrial animal movement, and forest carbon biomass in 300 m cells across Ontario. Color scales are based on quantile intervals (in 20% increments). Dark brown regions (top right corner of color matrix) represent cells that are in the top 20% quantile for both current density and carbon layers. Solid black lines show the boundaries of selected large (>150,000 ha) protected areas (excluding marine protected and conserved areas) and light blue polygons depict large waterbodies (>100 km^2^). White spaces in the map represent areas where we have current density values, but forest carbon values are missing.

**FIGURE 4:**
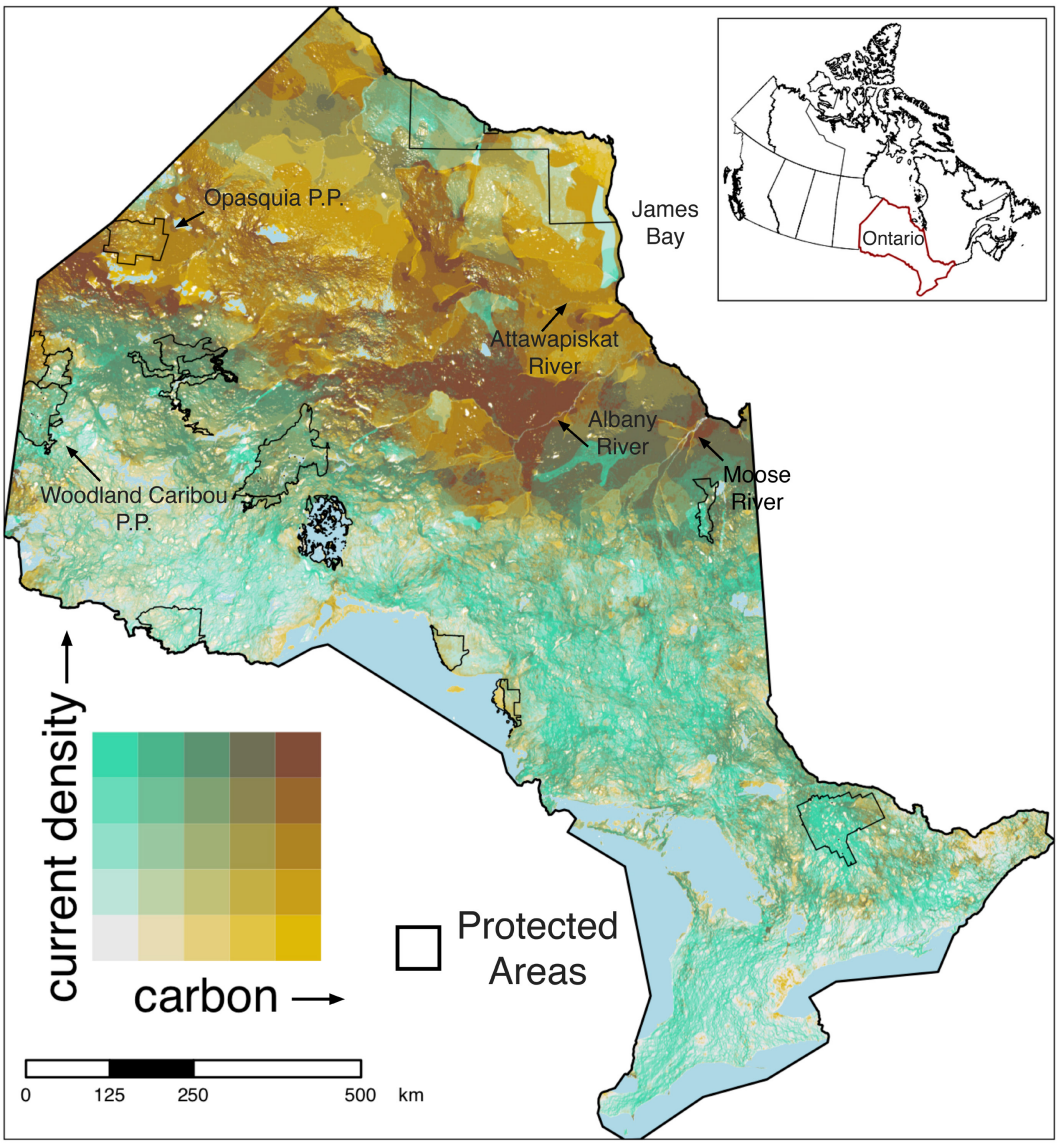
Bivariate map showing spatial overlap between current density, representing the probability of animal movement, and soil organic carbon biomass in 300 m cells across Ontario. Color scales are based on quantile intervals (in 20% increments). Dark brown regions (top right corner of color matrix) represent cells that are in the top 20% quantile for both current density and carbon layers. Solid black lines show the boundaries of selected large (>150,000 ha) protected areas (excluding marine protected and conserved areas) and light blue polygons depict large waterbodies (>100 km^2^). White spaces in the map represent areas where we have current density values, but soil carbon values are missing.

Scatterplots of current density values against both forest and soil carbon values in Ontario displayed weak positive relationships between connectivity and carbon storage (Figures 1 and 2; Spearman rank correlation of forest carbon and current density: rho = 0.074, *p* < .001; correlation of soil carbon and current density: rho = 0.093, *p* < .001). Our bivariate maps revealed varying amounts of spatial overlap between current density and carbon in Ontario, depending on the location and the type of carbon stock. The spatial distribution of hotspots between current density and carbon varied for forest carbon (Figure 3) and soil organic carbon (Figure 4). There was low overlap of highest quantile areas between connectivity and forest carbon (i.e., 4.6% or 49,597.7 km^2^ of Ontario consisted of overlapping highest quantile areas, or hotspots) and only 3.4% (37,148.8 km^2^) between connectivity and soil carbon. Further, there was only a 2% (1747 km^2^) overlap between hotspots identified by the connectivity‐forest carbon map (Figure 3) and the connectivity‐soil carbon map (Figures 4, S1), which corresponds to 0.16% of Ontario.

**FIGURE 5:**
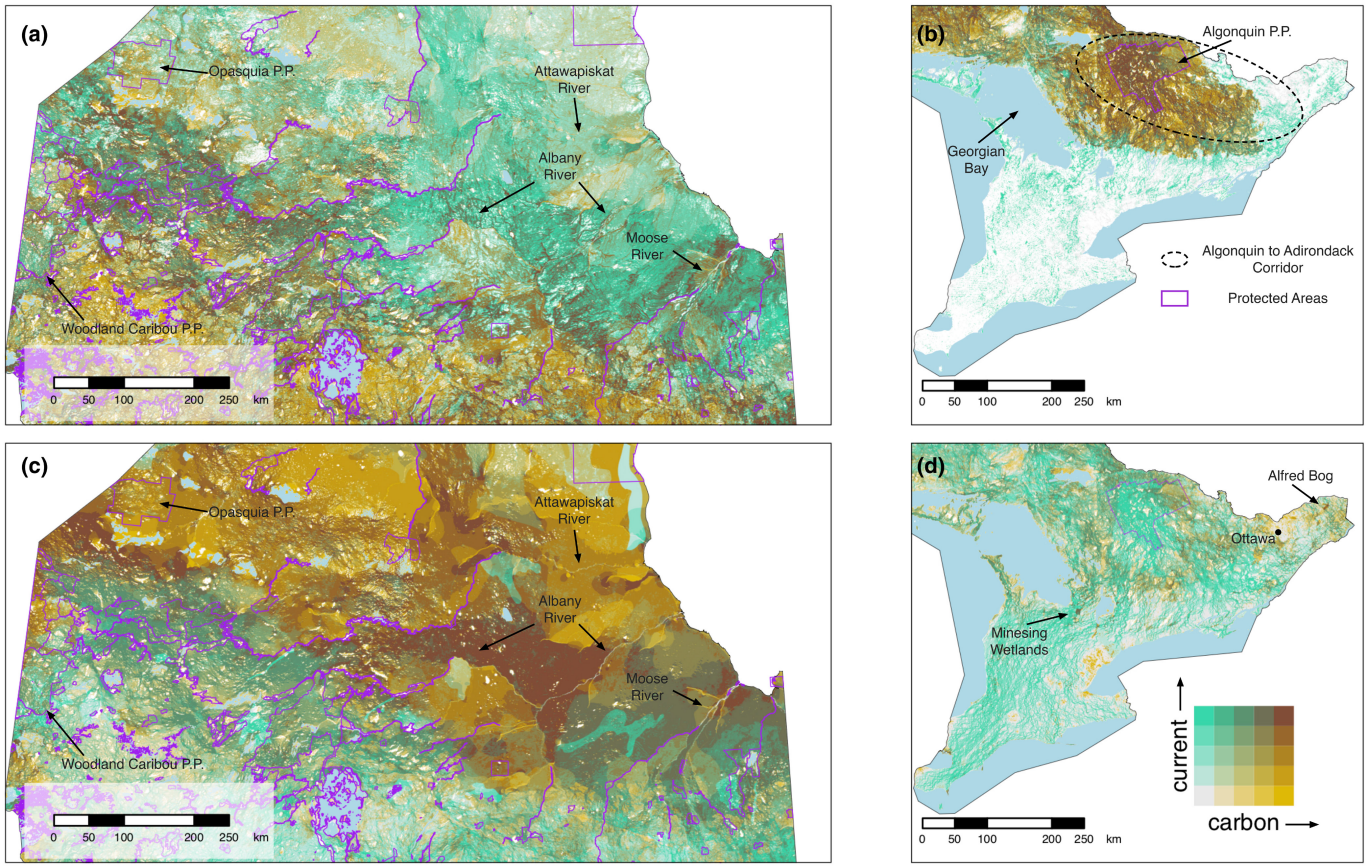
Vignettes to highlight hotspots for current density, representing the probability of animal movement and: (a) forest carbon in northern Ontario, (b) forest carbon in southern Ontario, (c) soil carbon in northern Ontario, and (d) soil carbon in southern Ontario. Purple lines show the boundaries of protected and conserved areas in Ontario, the dashed oval represents the general extent of the Algonquin to Adirondacks corridor, and light blue polygons show large waterbodies (>100 km^2^). Protected areas boundaries, except Algonquin Provincial Park, were excluded from panels (b) and (d) to maintain map clarity.

Areas most important for both connectivity and forest carbon storage were largely found within the northern regions of the province, which coincided with the Boreal Shield ecozone of Canada. For example, prominent areas of overlap occurred around Algonquin Provincial Park, north of Lake Superior, and moving northwest from Lake Nipigon into eastern Manitoba (Figure 3). Fewer hotspots were evident farther north in Ontario; however, important areas did appear along rivers south of James and Hudson Bay, such as the Moose and Albany rivers (Figures 3, 5a). In the south, hotspots for connectivity and forest carbon were concentrated in the area east of Algonquin Park toward the Canada‐US border, which is part of the existing Algonquin to Adirondacks (A2A) connectivity initiative (Figure 5b). Our analysis did not identify any hotspots at the provincial extent in the southern region of the province; however, important corridors still exist in the southwest despite the high degree of anthropogenic disturbance. For example, areas of high current density can be seen along a north–south corridor east of Georgian Bay (Figure 5b). These areas of high connectivity and low carbon storage may be important to protect to maintain connectivity and provide good opportunities for restoration (e.g., tree planting) to enhance both connectivity and carbon stocks.

Hotspots for connectivity and soil carbon storage covered a large area across Ontario (~3.7 million hectares total) with many of these areas occurring throughout the Hudson Bay Lowlands in northern Ontario. The most important areas were found south of James Bay around the mouth of the Moose River, between the Albany and Attawapiskat Rivers, and nearing the Ontario‐Manitoba border between Woodland Caribou and Opasquia Provincial Parks (Figures 4 and 5c). These areas are particularly important as they were also identified as important areas for connectivity and forest carbon. Many smaller hotspots (top 20% values for both layers) were also identified throughout the north as well as large areas where top 20% values for one layer overlap with top 40% values of another. Similarly, several smaller areas appear in southern Ontario where top 20% values for current density overlap with top 40% values for soil carbon (e.g., Minesing wetlands and Alfred Bog; Figure 5d).

**FIGURE 6:**
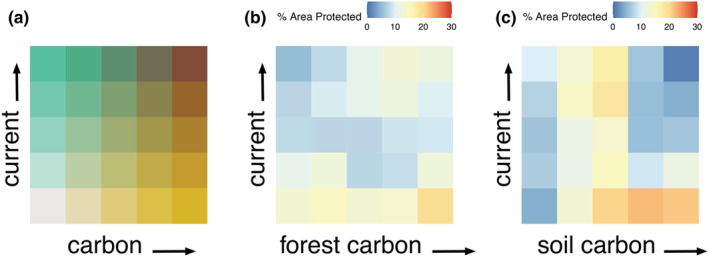
Color matrices displaying (a) the underlying layout for the spatial overlap of current density and carbon layers with cell values divided in 20% quantile intervals; the percent area within each quantile group contained within current protected areas for bivariate maps of current density and (b) forest carbon and (c) soil organic carbon.

Our analysis indicates that the current system of protected areas in Ontario protects 12% (5970.2 km^2^) of hotspots for connectivity and forest carbon and only 1% (402.5 km^2^) of hotspots for connectivity and soil carbon (Figure 6). The highest percentage of protected areas coverage in Ontario was found in areas most important for carbon, but least important for connectivity, at 19.7% (2725.6 km^2^) and 22.2% (4097.4 km^2^), respectively (Figure 6). That is, the existing system of protected areas tend to protect areas with high carbon storage, but low current density.

## 4 DISCUSSION

We found a low degree of geographic overlap between the highest quantile areas for connectivity and forest carbon (4.6%) and between connectivity and soil carbon (3.4%). We also found overall connectivity to be weakly correlated with both forest and soil carbon storage. This is consistent with other studies reporting a weak correlation between carbon and biodiversity (Di Marco et al., 2018) and climate refugia (Carroll & Ray, 2021). We predicted that areas important for carbon storage and connectivity would be within large intact landscapes. Although pixels with high carbon storage values were often within large, intact areas with low movement costs, this did not always translate into high current densities, largely due to the influence of geometry and the tendency for proximate high‐cost areas to lead to a funneling of current flow (Marrotte et al., 2017).

The limited spatial alignment of the highest quantiles for carbon and connectivity suggest that the hotspots where the top 20% for both measures overlap may be priorities for protection. Further, we found there was only a 2% overlap between hotspots identified between the two maps (i.e., hotspots for connectivity, forest carbon, and soil carbon). These overlapping areas between the two maps (Figures 3 and 4) were all found in the naturally intact landscapes of northern Ontario: south of Opasquia Provincial Park at the Ontario‐Manitoba border, along the Albany River, and at the mouth of the Moose River (Figure S1). The limited overlap between these three metrics indicates that these areas are high priority for protection and that conservation strategies with an objective to incorporate both connectivity and carbon should consider forest carbon and soil carbon separately. Including only one or the other likely would result in areas of importance being missed, while pooling both together would lead to a bias toward regions important for soil carbon storage driven by the greater magnitude of carbon stored in soils (Crowther et al., 2019). Although protecting areas important for soil carbon alone (e.g., peatlands) would undoubtedly conserve large amounts of stored carbon, disregarding forest carbon sequestration and storage would limit the near‐term carbon mitigation potential of Ontario's forests (Chen et al., 2018). It would also lessen the biodiversity benefits from protecting ecologically representative areas (Aichi Target 11). Further, while we found limited overlap between hotspots for carbon storage and connectivity, highlighting the critical need to protect these areas, we also note that hotspots for carbon or connectivity alone are still extremely valuable and more widespread. Given the current level of protected areas in Ontario, these areas should also be considered priorities for protection or restoration.

Ontario's soil carbon stocks are largely contained within the vast boreal peatlands of the Hudson Bay Lowlands (Harris et al., 2021; Sothe et al., 2022) which is also where our bivariate maps reveal strong spatial congruence between landscape connectivity and soil carbon storage. These carbon stores have been identified as irrecoverable at a global scale meaning they would not be recoverable within the timeframe necessary to reach net‐zero (Goldstein et al., 2020; Noon et al., 2021; Packalen et al., 2014). As predicted, the largest areas of overlap occurred in the northern part of the province where boreal forest and peatland ecosystems remain largely intact. Protection of these hotspot areas would make a considerable contribution toward climate change targets while enhancing biodiversity. Further, across northern Canada, especially northern Ontario, there is a high degree of overlap between Indigenous managed lands and territories and important carbon sinks (Artelle et al., 2019; Townsend et al., 2020); most of the hotspots identified by our analysis follow this trend. Thus, identification and design of protected areas in these regions should occur in collaboration with Indigenous communities/governments, be guided by Indigenous expertise, and be based on the priorities of Indigenous Governments (Indigenous Circle of Experts, 2018). Moreover, protection of these hotspots may best be served through the identification of new Indigenous Protected and Conserved Areas (IPCAs; also referred to as Indigenous led area‐based conservation) which shift away from traditional concepts and governance of protected areas; they are led by Indigenous communities and integrate culture, language, socio‐economic factors, and traditional land use as part of conservation efforts (Indigenous Circle of Experts, 2018). Mapping resources such as we present here could be helpful for Indigenous governments to advance IPCA efforts.

Hotspots of soil carbon and connectivity appear in the region between the Albany and Attawapiskat rivers where a large east–west corridor coincides with these large, intact peatlands. Not surprisingly, this corridor has been shown to be critical to migrating species such as woodland caribou (OMECP, 2020; OMNRF, 2019) and birds (ECCC, 2013) and this area may provide a natural buffer or refugia for the persistence of biodiversity as the climate changes (Morelli et al., 2020; Stralberg, Arseneault, et al., 2020; Stralberg, Carroll, & Nielsen, 2020). Our maps also identify hotspots for both forest and soil carbon with connectivity at the western border of Ontario and Manitoba. Protection here would not only maximize carbon storage benefits (i.e., protecting important forest and soil stocks), but would also contribute to global biodiversity goals of creating well‐connected networks of protected areas (Aichi Target 11) by connecting Woodland Caribou Provincial Park to the south with Opasquia Provincial Park to the north.

Isolation of Ontario's north has safeguarded these hotspots to date; however, soil and peat stocks are increasingly threatened by land‐use and land cover changes, resulting from mineral extraction, draining for agriculture, peat extraction, and shrubification, for example (Harris et al., 2021). Due to the likely irrecoverability of soil carbon in this region, its importance to wildlife, and the potential to support biodiversity in the future warmer climate, protection of these hotspots could be critically important while also being cost‐effective (Cook‐Patton et al., 2021; Goldstein et al., 2020). Currently only ~1% of hotspots for connectivity and soil carbon are protected in Ontario, which conserves ~0.04 Pg of carbon. Increasing protection in this region to even 5% has the potential to conserve ~0.2 Pg of carbon, which is equivalent to CO_2_ emissions from 188 coal‐fired power plants for a year (USEPA, 2015). This represents a 400% increase in carbon protected, while also helping to maintain connectivity between Canada's eastern and western provinces (Murray et al., 2017). Given the high degree of overlap between Indigenous territories and carbon sinks (Townsend et al., 2020) and the importance of Indigenous managed‐lands as habitat for biodiversity (Schuster et al., 2019), these northern hotspots may be particularly important candidates for IPCAs that are led by Indigenous communities and integrate Indigenous culture and priorities. Importantly, any proposal of new protected areas should uphold inherent Indigenous rights through an open partnership with Indigenous peoples and governments and should help advance Indigenous‐led conservation (Indigenous Circle of Experts, 2018).

Forest carbon makes up a smaller proportion of carbon stocks in Ontario. Forest carbon is generally considered recoverable carbon due to natural regeneration, reforestation activities, and the ability to store carbon long‐term in harvested wood products. Our bivariate maps reveal areas with strong spatial congruence between landscape connectivity and forest carbon storage throughout the boreal shield ecozone of Ontario. Identified hotspots for connectivity and forest carbon may face more immediate threats from resource extraction (e.g., logging and mining), forest fires, pest outbreaks, extreme drought, and invasive species (Wells et al., 2020), with the latter four likely to increase as a consequence of climate change (Allen et al., 2015; Anderegg et al., 2020; Price et al., 2013) Ontario has some of the largest intact boreal forests in Canada (Watson et al., 2018), but deforestation has occurred both within forestry tenures (OMNRF, 2021; Smith & Cheng, 2016) as well as outside of managed forests. While there are threats facing forest carbon stocks, the significance of soil carbon stocks in the north compared with forest stocks clearly expresses the disproportionately important role of soil carbon in climate change mitigation and the critical need to protect these stocks. Protection and management of these different carbon pools may therefore require different strategies.

The exceptional value of intact forests for carbon storage and biodiversity is generally noted meaning that protection of these areas is important (Watson et al., 2018). The recoverable nature of forests and their broad utility for connectivity could be more compatible with a combination of protection and improved forest management practices (Malcolm et al., 2020). For example, an analysis of natural climate solutions in Canada found improved forest management (e.g., a combination of old growth conservation and regeneration after harvest) to be the fourth largest opportunity for climate change mitigation potential in 2030 (Drever et al., 2021). Aside from national and provincial protected and conserved areas, those areas that are managed in ways that support biodiversity and climate even when not the primary objective (i.e., other effective area‐based conservation measures or OECMs) may be particularly effective at conserving and increasing connectivity in managed landscapes, such as forests and farmlands (Dudley et al., 2018; Gurney et al., 2021; Maxwell et al., 2020). For example, permanently set aside areas within managed forests, such as old growth stands, high‐biodiversity areas, or high‐value connectivity areas may help to contribute to long‐term biodiversity conservation and climate change mitigation goals (IUCN, 2018), especially if combined with sustainable management of the surrounding forests (Aichi Target 7).

In the southern part of the province, we identified spatial congruence between forest carbon and connectivity coinciding with the known Algonquin to Adirondacks Corridor (A2A). Addition of protected and conserved areas within this corridor could provide significant benefits to the transboundary movement of wildlife between Algonquin Park in Ontario, Canada, and Adirondack Park in New York State, USA. Additionally, Proctor et al. (2022) found that protection of species at risk habitat in Ontario may be most cost effective in the central region of the province where land cost is low, but species at risk richness is still relatively high. This aligns with areas important for connectivity and forest carbon storage identified by our analysis. Moreover, the eastern section of A2A and regions of southwestern Ontario exhibit importance for connectivity but are lacking in forest carbon stocks. These regions could therefore be an important focus of ecological restoration efforts (e.g., tree‐planting initiatives, grassland restoration). Restoring degraded ecosystems and corridors is an imperative step to enhancing biodiversity and can increase both above‐ and below‐ground carbon stocks (Gunn et al., 2019; Soto‐Navarro et al., 2020; Valach et al., 2021). In this case, restoring within the A2A could help maintain connectivity, while also building carbon stores. Our maps could also be used to identify other areas where carbon stocks are high and protection could reduce their vulnerability to loss or likewise, where carbon is low and restoration activities, such as described above, could increase these values over time.

Our maps identified Catchacoma Forest, an old growth eastern hemlock (*Tsuga canadensis*) forest northeast of Lake Simcoe, as an area of interest for connectivity and forest carbon. In addition, Catchacoma Forest has been identified as the largest known old growth eastern hemlock stand in Canada and an important site for various species at risk (Quinby, 2020); however, it is threatened by the invasive hemlock woolly adelgid (NRCAN, 2015) and is an actively logged area. We also identified small hotspots for connectivity and soil carbon in the south. Of particular interest are Minesing Wetlands and Alfred Bog, which represent some of the last large wetlands in southern Ontario. The Minesing Wetlands are currently being assessed as a Key Biodiversity Area, which further supports it as a priority area for protection. Similarly, Alfred Bog is a provincially significant wetland, an Area of Natural and Scientific Interest, has protection through various NGOs, and is in the process of Provincial Park designation (NCC, 2021). Protection of these provincially significant wetlands would provide multiple benefits for climate change mitigation, biodiversity, human wellbeing, as well as other important ecosystem services.

Interestingly, the results of our provincial analysis exhibit similarities with recent global analyses. Soto‐Navarro et al. (2020) assessed the spatial overlap of global carbon stores with measures of reactive (vulnerability) and proactive (intactness) biodiversity. Similarly, Dinerstein et al. (2020) mapped global conservation priorities for biodiversity and climate stabilization as well as identified potential wildlife corridors between current protected areas. All three studies identify priority areas within the Hudson Bay Lowlands and Boreal Shield in Ontario, with few areas of importance occurring in southern Ontario. The high resolution of our analyses, however, allowed us to distinguish more fine‐scale patterns of spatial overlap between carbon storage and connectivity, which will be useful for land‐use planners across different jurisdictions. Moreover, the separation of forest and soil carbon in our study illustrated the differences in conservation priorities identified when assessing the relationship of these different carbon pools with landscape connectivity. Still, it is apparent from the current and previous studies that the Hudson Bay Lowlands is a hotspot for carbon storage, connectivity, and biodiversity (Dinerstein et al., 2020; Soto‐Navarro et al., 2020) and is therefore a high priority for protection.

Existing protected and conserved areas in Ontario cover 10.7% of the province's area (Ontario Parks, 2021). Our analysis indicates that 12% of hotspots (top 20% overlap) for connectivity and forest carbon and only 1% of hotspots for connectivity and soil carbon are contained within current protected areas. A lack of protection was also evident for hotspots of biodiversity and carbon globally (Soto‐Navarro et al., 2020) and in Asia (Zhu et al., 2021). Similarly, Proctor et al. (2022) found that 50% of species at risk in Ontario had less than 10% of their habitat protected by existing protected areas. A recent temporal analysis by Maxwell et al. (2020) revealed that expansion of protected areas (between 2010 and 2019) made minimal contributions to conserving various elements of biodiversity and ecosystem services, including carbon storage and connectivity. These findings stress the critical need for resources such as the above‐mentioned studies and our current study for helping to guide the expansion of effective protected and conserved areas. Our integration of spatial data on carbon storage and connectivity provides a framework to consider synergies between climate change mitigation and biodiversity conservation, such that we were able to identify key areas for targeted NbS strategies in Ontario.

We believe our maps will be useful for land‐use planners and stakeholders in identifying areas important for expanding protected areas; however, we acknowledge several caveats. We used the most up to date carbon maps available but there remain uncertainties associated with these carbon stock estimates, particularly in peatland soils (Sothe et al., 2022). Second, there may be uncertainty with how well the connectivity map we used predicts areas important for connectivity in northern Ontario given that much of the mammal data used to validate the map was from western Canada. Finally, while we would have ideally included data layers for carbon, connectivity, and biodiversity, the KBA data for Ontario was not yet available, however our maps still identified areas that coincided with designated and potential KBAs. Therefore, we suggest that future analyses could update our maps with biodiversity data, especially KBA locations as they become available. Despite these caveats, we consider that our maps accurately identify hotspots for connectivity and carbon storage in Ontario and can be a valuable resource for the identification of new protected and conserved areas and sites for ecological restoration.

## 5 CONCLUSION

Global biodiversity and climate targets will require ambitious national and regional actions to protect and sustainably manage remaining areas of high ecological integrity and restore damaged and degraded areas to improve biodiversity. We intersected high‐resolution spatial layers for carbon storage and landscape connectivity to provide a framework for exploring synergies between climate change mitigation and biodiversity conservation in Ontario, Canada. We found a weak relationship between these metrics; however, the maps we produced still revealed hotspots with high values for both connectivity and carbon storage in the province. Our results align with previous global analyses, providing further support that the areas identified are priorities for protection. We believe our high‐resolution mapping resources will be useful for land‐use planners and policymakers to identify areas important for nature‐based climate solutions. Further, our methods could easily be adopted by other provinces, territories, and other jurisdictions to contribute to recent ambitious Canadian national targets to reach 30% protection of terrestrial lands by 2030. Critical to achieving the ambitious 30 × 30 target is not only increasing coverage, but also effectiveness of protected areas. Our approach can help to maximize the co‐benefits for climate change mitigation and biodiversity conservation.

## AUTHOR CONTRIBUTIONS

**Paul O'Brien:** Data curation (lead); formal analysis (lead); methodology (equal); visualization (lead); writing – original draft (equal); writing – review and editing (equal). **John S. Gunn:** Methodology (equal); writing – original draft (equal); writing – review and editing (equal). **Alison Clark:** Conceptualization (equal); writing – original draft (equal); writing – review and editing (equal). **Jenny Gleeson:** Conceptualization (equal); writing – original draft (equal); writing – review and editing (equal). **Richard Pither:** Writing – original draft (equal); writing – review and editing (equal). **Jeff Bowman:** Conceptualization (equal); methodology (equal); writing – original draft (equal); writing – review and editing (equal).

## CONFLICT OF INTEREST

The authors declare no conflict of interest.

## Supporting information


Appendix S1:

## Data Availability

Current Density Map and Movement Cost Layer (https://osf.io/z2qs3/; DOI https://doi.org/10.17605/OSF.IO/Z2QS3). Forest Carbon Stock Map (https://doi.org/10.4121/14572929.v1) and Soil Carbon Stock Map (https://doi.org/10.4121/16686154.v3). Our output Forest Carbon‐Connectivity and Soil Carbon‐Connectivity Maps (https://doi.org/10.6084/m9.figshare.21725435).

## References

[ece39725-bib-0001] Allen, C. D. , Breshears, D. D. , & McDowell, N. G. (2015). On underestimation of global vulnerability to tree mortality and forest die‐off from hotter drought in the Anthropocene. Ecosphere, 6(8), 1–55. 10.1890/ES15-00203.1

[ece39725-bib-0002] Anderegg, W. R. L. , Trugman, A. T. , Badgley, G. , Anderson, C. M. , Bartuska, A. , Ciais, P. , Cullenward, D. , Field, C. B. , Freeman, J. , Goetz, S. J. , Hicke, J. A. , Huntzinger, D. , Jackson, R. B. , Nickerson, J. , Pacala, S. , & Randerson, J. T. (2020). Climate‐driven risks to the climate mitigation potential of forests. Science, 368(6497), eaaz7005. 10.1126/science.aaz7005 32554569

[ece39725-bib-0003] Arneth, A. , Shin, Y. J. , Leadley, P. , Rondinini, C. , Bukvareva, E. , Kolb, M. , Midgley, G. F. , Oberdorff, T. , Palomo, I. , & Saito, O. (2020). Post‐2020 biodiversity targets need to embrace climate change. Proceedings of the National Academy of Sciences of the United States of America, 117(49), 30882–30891. 10.1073/pnas.2009584117 33288709 PMC7739876

[ece39725-bib-0004] Artelle, K. A. , Zurba, M. , Bhattacharyya, J. , Chan, D. E. , Brown, K. , Housty, J. , & Moola, F. (2019). Supporting resurgent indigenous‐led governance: A nascent mechanism for just and effective conservation. Biological Conservation, 240, 108284. 10.1016/j.biocon.2019.108284

[ece39725-bib-0005] Barnett, K. , & Belote, R. T. (2021). Modeling an aspirational connected network of protected areas across North America. Ecological Applications, 31(6), e02387. 10.1002/eap.2387 34137106 PMC8459232

[ece39725-bib-0006] Busch, J. , & Grantham, H. S. (2013). Parks versus payments: Reconciling divergent policy responses to biodiversity loss and climate change from tropical deforestation. Environmental Research Letters, 8(3), 034028. 10.1088/1748-9326/8/3/034028

[ece39725-bib-0007] Carroll, C. , & Ray, J. C. (2021). Maximizing the effectiveness of national commitments to protected area expansion for conserving biodiversity and ecosystem carbon under climate change. Global Change Biology, 27(15), 3395–3414. 10.1111/gcb.15645 33852186 PMC8360173

[ece39725-bib-0008] Chen, J. , Ter‐Mikaelian, M. T. , Ng, P. Q. , & Colombo, S. J. (2018). Ontario's managed forests and harvested wood products contribute to greenhouse gas mitigation from 2020 to 2100. The Forestry Chronicle, 94(3), 269–282. 10.5558/tfc2018-040

[ece39725-bib-0009] Cohen‐Shacham, E. , Andrade, A. , Dalton, J. , Dudley, N. , Jones, M. , Kumar, C. , Maginnis, S. , Maynard, S. , Nelson, C. R. , Renaud, F. G. , Welling, R. , & Walters, G. (2019). Core principles for successfully implementing and upscaling nature‐based solutions. Environmental Science & Policy, 98, 20–29. 10.1016/j.envsci.2019.04.014

[ece39725-bib-0010] Cohen‐Shacham, E. , Walters, G. , Janzen, C. , & Maginnis, S. (2016). Nature‐based solutions to address global societal challenges. Gland, Switzerland.

[ece39725-bib-0011] Cook‐Patton, S. C. , Drever, C. R. , Griscom, B. W. , Hamrick, K. , Hardman, H. , Kroeger, T. , Pacheco, P. , Raghav, S. , Stevenson, M. , Webb, C. , Yeo, S. , & Ellis, P. W. (2021). Protect, manage and then restore lands for climate mitigation. Nature Climate Change, 11(12), 1027–1034. 10.1038/s41558-021-01198-0

[ece39725-bib-0012] Crowther, T. W. , van den Hoogen, J. , Wan, J. , Mayes, M. A. , Keiser, A. D. , Mo, L. , Averill, C. , & Maynard, D. S. (2019). The global soil community and its influence on biogeochemistry. Science, 365(6455), eaav0550. 10.1126/science.aav0550 31439761

[ece39725-bib-0013] Di Marco, M. , Watson, J. E. M. , Currie, D. J. , Possingham, H. P. , & Venter, O. (2018). The extent and predictability of the biodiversity–carbon correlation. Ecology Letters, 21(3), 365–375. 10.1111/ele.12903 29314473

[ece39725-bib-0014] Dickson, B. G. , Albano, C. M. , Anantharaman, R. , Beier, P. , Fargione, J. , Graves, T. A. , Gray, M. E. , Hall, K. R. , Lawler, J. J. , Leonard, P. B. , Littlefield, C. E. , McClure, M. L. , Novembre, J. , Schloss, C. A. , Schumaker, N. H. , Shah, V. B. , & Theobald, D. M. (2019). Circuit‐theory applications to connectivity science and conservation. Conservation Biology, 33(2), 239–249. 10.1111/cobi.13230 30311266 PMC6727660

[ece39725-bib-0015] Dinerstein, E. , Joshi, A. R. , Vynne, C. , Lee, A. T. L. , Pharand‐Deschênes, F. , França, M. , Fernando, S. , Birch, T. , Burkart, K. , Asner, G. P. , & Olson, D. (2020). A “global safety net” to reverse biodiversity loss and stabilize earth's climate. Science Advances, 6(36), eabb2824. 10.1126/sciadv.abb2824 32917614 PMC7473742

[ece39725-bib-0016] Doyle, P. G. , & Snell, J. L. (1984). Random walks and electric networks (p. 159). Mathematical Association of America.

[ece39725-bib-0017] Drever, C. R. , Cook‐Patton, S. C. , Akhter, F. , Badiou, P. H. , Chmura, G. L. , Davidson, S. J. , Desjardins, R. L. , Dyk, A. , Fargione, J. E. , Fellows, M. , Filewod, B. , Hessing‐Lewis, M. , Jayasundara, S. , Keeton, W. S. , Kroeger, T. , Lark, T. J. , Le, E. , Leavitt, S. M. , LeClerc, M. E. , … Kurz, W. A. (2021). Natural climate solutions for Canada. Science Advances, 7(23), eabd6034. 10.1126/sciadv.abd6034 34088658 PMC8177698

[ece39725-bib-0018] Dudley, N. , Jonas, H. , Nelson, F. , Parrish, J. , Pyhälä, A. , Stolton, S. , & Watson, J. E. M. (2018). The essential role of other effective area‐based conservation measures in achieving big bold conservation targets. Glob. Ecol. Conserv., 15, e00424. 10.1016/j.gecco.2018.e00424

[ece39725-bib-0019] ECCC . (2013). Bird conservation strategy for bird conservation region 7 in Ontario: Taiga Shield and Hudson Plains. ECCC.

[ece39725-bib-0020] ECCC . (2020). Canada joins the high ambition coalition for nature and people. ECCC.

[ece39725-bib-0021] ECCC . (2021). Canadian protected and conserved areas database. ECCC.

[ece39725-bib-0022] Goldstein, A. , Turner, W. R. , Spawn, S. A. , Anderson‐Teixeira, K. J. , Cook‐Patton, S. , Fargione, J. , Gibbs, H. K. , Griscom, B. , Hewson, J. H. , Howard, J. F. , Ledezma, J. C. , Page, S. , Koh, L. P. , Rockström, J. , Sanderman, J. , & Hole, D. G. (2020). Protecting irrecoverable carbon in Earth's ecosystems. Nature Climate Change, 10(4), 287–295. 10.1038/s41558-020-0738-8

[ece39725-bib-0023] Government of Canada . (2022). 2030 emissions reduction plan: Clean air, strong economy. Government of Canada.

[ece39725-bib-0024] Grantham, H. S. , Duncan, A. , Evans, T. D. , Jones, K. R. , Beyer, H. L. , Schuster, R. , Walston, J. , Ray, J. C. , Robinson, J. G. , Callow, M. , Clements, T. , Costa, H. M. , DeGemmis, A. , Elsen, P. R. , Ervin, J. , Franco, P. , Goldman, E. , Goetz, S. , Hansen, A. , … Watson, J. E. M. (2020). Anthropogenic modification of forests means only 40% of remaining forests have high ecosystem integrity. Nature Communications, 11(1), 5978. 10.1038/s41467-020-19493-3 PMC7723057 33293507

[ece39725-bib-0025] Gunn, J. S. , Ducey, M. J. , & Belair, E. (2019). Evaluating degradation in a north American temperate forest. Forest Ecology and Management, 432, 415–426. 10.1016/j.foreco.2018.09.046

[ece39725-bib-0026] Gurney, G. G. , Darling, E. S. , Ahmadia, G. N. , Agostini, V. N. , Ban, N. C. , Blythe, J. , Claudet, J. , Epstein, G. , Estradivari, A. , Jonas, H. D. , Armitage, D. , Campbell, S. J. , Cox, C. , Friedman, R. , Gill, D. , Lestari, P. , Mangubhai, S. , McLeod, E. , Muthiga, N. A. , … Jupiter, S. D. (2021). Biodiversity needs every tool in the box: Use OECMs. Nature, 595(7869), 646–649. 10.1038/d41586-021-02041-4 34312552

[ece39725-bib-0027] Hall, K. R. , Anantharaman, R. , Landau, V. A. , Clark, M. , Dickson, B. G. , Jones, A. , Platt, J. , Edelman, A. , & Shah, V. B. (2021). Circuitscape in julia: Empowering dynamic approaches to connectivity assessment. Landscape, 10(3), 301. 10.3390/land10030301

[ece39725-bib-0028] Harris, L. I. , Richardson, K. , Bona, K. A. , Davidson, S. J. , Finkelstein, S. A. , Garneau, M. , McLaughlin, J. , Nwaishi, F. , Olefeldt, D. , Packalen, M. , Roulet, N. T. , Southee, F. M. , Strack, M. , Webster, K. L. , Wilkinson, S. L. , & Ray, J. C. (2021). The essential carbon service provided by northern peatlands. Frontiers in Ecology and the Environment, 20(4), 222–230. 10.1002/fee.2437

[ece39725-bib-0029] Heller, N. E. , & Zavaleta, E. S. (2009). Biodiversity management in the face of climate change: A review of 22 years of recommendations. Biological Conservation, 142(1), 14–32. 10.1016/j.biocon.2008.10.006

[ece39725-bib-0030] Hirsh‐Pearson, K. , Johnson, C. J. , Schuster, R. , Wheate, R. D. , & Venter, O. (2022). Canada's human footprint reveals large intact areas juxtaposed against areas under immense anthropogenic pressure. Facets, 7, 398–419.

[ece39725-bib-0031] Holl, K. D. , & Brancalion, P. H. S. (2020). Tree planting is not a simple solution. Science, 368(6491), 580–581. 10.1126/science.aba8232 32381704

[ece39725-bib-0032] Hooftman, D. A. P. , Edwards, B. , & Bullock, J. M. (2016). Reductions in connectivity and habitat quality drive local extinctions in a plant diversity hotspot. Ecography, 39(6), 583–592. 10.1111/ecog.01503

[ece39725-bib-0033] Indigenous Circle of Experts . (2018). We Rise Together: Achieving Pathway to Canada Target 1 through the creation of Indigenous Protected and Conserved Areas in the spirit and practice of reconciliation.

[ece39725-bib-0034] IPBES . (2019). Global assessment report on biodiversity and ecosystem services of the intergovernmental science‐policy platform on biodiversity and ecosystem services. IPBES.

[ece39725-bib-0035] IPCC . (2022a). Climate change 2022: Impacts, adaptation, and vulnerability (contribution of working group II to the sixth assessment report of IPCC). IPCC.

[ece39725-bib-0036] IPCC . (2022b). Climate change 2022: Mitigation of climate change. Contribution of Working Group III to the Sixth Assessment Report of the Intergovernmental Panel on Climate Change. IPCC.

[ece39725-bib-0037] IUCN . (2018). Recognising and reporting other effective area‐based conservation measures. IPCC. https://portals.iucn.org/library/sites/library/files/documents/PATRS‐003‐En.pdf

[ece39725-bib-0038] Krosby, M. , Breckheimer, I. , John Pierce, D. , Singleton, P. H. , Hall, S. A. , Halupka, K. C. , Gaines, W. L. , Long, R. A. , McRae, B. H. , Cosentino, B. L. , & Schuett‐Hames, J. P. (2015). Focal species and landscape “naturalness” corridor models offer complementary approaches for connectivity conservation planning. Landscape Ecology, 30(10), 2121–2132. 10.1007/s10980-015-0235-z

[ece39725-bib-0039] Kurz, W. A. , Shaw, C. H. , Boisvenue, C. , Stinson, G. , Metsaranta, J. , Leckie, D. , Dyk, A. , Smyth, C. , & Neilson, E. T. (2013). Carbon in Canada's boreal forest‐A synthesis. Environmental Reviews, 21(4), 260–292. 10.1139/er-2013-0041

[ece39725-bib-0040] Lewis, S. L. , Wheeler, C. E. , Mitchard, E. T. A. , & Koch, A. (2019). Restoring natural forests is the best way to remove atmospheric carbon. Nature, 568(7750), 25–28. 10.1038/d41586-019-01026-8 30940972

[ece39725-bib-0041] Locke, H. , Ellis, E. C. , Venter, O. , Schuster, R. , Ma, K. , Shen, X. , Woodley, S. , Kingston, N. , Bhola, N. , Strassburg, B. B. N. , Paulsch, A. , Williams, B. , & Watson, J. E. M. (2019). Three global conditions for biodiversity conservation and sustainable use: An implementation framework. National Science Review, 6(6), 1080–1082. 10.1093/nsr/nwz136 34691979 PMC8291457

[ece39725-bib-0042] Malcolm, J. R. , Holtsmark, B. , & Piascik, P. W. (2020). Forest harvesting and the carbon debt in boreal east‐Central Canada. Climatic Change, 161(3), 433–449. 10.1007/s10584-020-02711-8

[ece39725-bib-0043] Marchese, C. (2015). Biodiversity hotspots: A shortcut for a more complicated concept. Global Ecology Conservation, 3, 297–309. 10.1016/j.gecco.2014.12.008

[ece39725-bib-0044] Marrotte, R. R. , Bowman, J. , Brown, M. G. C. , Cordes, C. , Morris, K. Y. , Prentice, M. B. , & Wilson, P. J. (2017). Multi‐species genetic connectivity in a terrestrial habitat network. Movement Ecology, 5(1), 1–11. 10.1186/s40462-017-0112-2 29043084 PMC5629812

[ece39725-bib-0045] Maxwell, S. L. , Cazalis, V. , Dudley, N. , Hoffmann, M. , Rodrigues, A. S. L. , Stolton, S. , Visconti, P. , Woodley, S. , Kingston, N. , Lewis, E. , Maron, M. , Strassburg, B. B. N. , Wenger, A. , Jonas, H. D. , Venter, O. , & Watson, J. E. M. (2020). Area‐based conservation in the twenty‐first century. Nature, 586(7828), 217–227. 10.1038/s41586-020-2773-z 33028996

[ece39725-bib-0046] McRae, B. H. , & Beier, P. (2007). Circuit theory predicts gene flow in plant and animal populations. Proceedings of the National Academy of Sciences of the United States of America, 104(50), 19885–19890. 10.1073/pnas.0706568104 18056641 PMC2148392

[ece39725-bib-0047] Mitchell, M. G. E. , Schuster, R. , Jacob, A. L. , Hanna, D. E. L. , Dallaire, C. O. , Raudsepp‐Hearne, C. , Bennett, E. M. , Lehner, B. , & Chan, K. M. A. (2021). Identifying key ecosystem service providing areas to inform national‐scale conservation planning. Environmental Research Letters, 16(1), 014038. 10.1088/1748-9326/abc121

[ece39725-bib-0048] Morelli, T. L. , Barrows, C. W. , Ramirez, A. R. , Cartwright, J. M. , Ackerly, D. D. , Eaves, T. D. , Ebersole, J. L. , Krawchuk, M. A. , Letcher, B. H. , Mahalovich, M. F. , Meigs, G. W. , Michalak, J. L. , Millar, C. I. , Quiñones, R. M. , Stralberg, D. , & Thorne, J. H. (2020). Climate‐change refugia: Biodiversity in the slow lane. Frontiers in Ecology and the Environment, 18(5), 228–234. 10.1002/fee.2189 33424494 PMC7787983

[ece39725-bib-0049] Murray, D. L. , Peers, M. J. L. , Majchrzak, Y. N. , Wehtje, M. , Ferreira, C. , Pickles, R. S. A. , Row, J. R. , & Thornton, D. H. (2017). Continental divide: Predicting climatemediated fragmentation and biodiversity loss in the boreal forest. PLoS One, 12(5), e0176706. 10.1371/journal.pone.0176706 28505173 PMC5432165

[ece39725-bib-0050] NCC . (2021). Alfred Bog. NCC.

[ece39725-bib-0051] Nesshöver, C. , Assmuth, T. , Irvine, K. N. , Rusch, G. M. , Waylen, K. A. , Delbaere, B. , Haase, D. , Jones‐Walters, L. , Keune, H. , Kovacs, E. , Krauze, K. , Külvik, M. , Rey, F. , van Dijk, J. , Vistad, O. I. , Wilkinson, M. E. , & Wittmer, H. (2017). The science, policy and practice of nature‐based solutions: An interdisciplinary perspective. Sci. Total Environ., 579, 1215–1227. 10.1016/j.scitotenv.2016.11.106 27919556

[ece39725-bib-0052] Noon, M. L. , Goldstein, A. , Ledezma, J. C. , Roehrdanz, P. R. , Cook‐Patton, S. C. , Spawn‐Lee, S. A. , Wright, T. M. , Gonzalez‐Roglich, M. , Hole, D. G. , Rockström, J. , & Turner, W. R. (2021). Mapping the irrecoverable carbon in Earth's ecosystems. Nature Sustain, 5(1), 37–46. 10.1038/s41893-021-00803-6

[ece39725-bib-0053] Noss, R. F. , Dobson, A. P. , Baldwin, R. , Beier, P. , Davis, C. R. , Dellasala, D. A. , Francis, J. , Locke, H. , Nowak, K. , Lopez, R. , Reining, C. , Trombulak, S. C. , & Tabor, G. (2012). Bolder thinking for conservation. Conservation Biology, 26(1), 1–4. 10.1111/j.1523-1739.2011.01738.x 22280321

[ece39725-bib-0054] NRCAN . (2015). Alien, invasive hemlock woolly adelgid found in Ontario. NRCAN.

[ece39725-bib-0055] OMECP . (2020). Woodland Caribou recovery strategy. OMNRF.

[ece39725-bib-0056] OMNRF . (2019). Woodland Caribou and the far north. NCC.

[ece39725-bib-0057] OMNRF . (2021). State of Ontario's Natural Resources – Forestry 2021. OMNRF.

[ece39725-bib-0058] Ontario Parks . (2021). State of Ontario's protected areas indicator report. Ontario Parks.

[ece39725-bib-0059] Packalen, M. S. , Finkelstein, S. A. , & McLaughlin, J. W. (2014). Carbon storage and potential methane production in the Hudson Bay lowlands since mid‐Holocene peat initiation. Nature Communications, 5, 4078. 10.1038/ncomms5078 24916043

[ece39725-bib-0060] Phillips, P. , Clark, M. M. , Baral, S. , Koen, E. L. , & Bowman, J. (2021). Comparison of methods for estimating omnidirectional landscape connectivity. Landscape Ecology, 36(6), 1647–1661. 10.1007/s10980-021-01254-2

[ece39725-bib-0061] Pither, R. , O'Brien, P. , Brennan, A. , Hirsh‐Pearson, K. , & Bowman, J. (2021). Areas important for ecological connectivity throughout Canada. bioRxiv, 2021.12.14.472649. 10.1101/2021.12.14.472649 PMC9946242 36812251

[ece39725-bib-0062] Poley, L. G. , Schuster, R. , Smith, W. , & Ray, J. C. (2022). Identifying differences in roadless areas in Canada based on global, national, and regional road datasets. Conservation Science and Practice, 4(4), e12656. 10.1111/csp2.12656

[ece39725-bib-0063] Price, D. T. , Alfaro, R. I. , Brown, K. J. , Flannigan, M. D. , Fleming, R. A. , Hogg, E. H. , Girardin, M. P. , Lakusta, T. , Johnston, M. , McKenney, D. W. , Pedlar, J. H. , Stratton, T. , Sturrock, R. N. , Thompson, I. D. , Trofymow, J. A. , & Venier, L. A. (2013). Anticipating the consequences of climate change for Canada's boreal forest ecosystems. Environmental Reviews, 21(4), 322–365. 10.1139/er-2013-0042

[ece39725-bib-0064] Proctor, C. A. , Schuster, R. , Buxton, R. T. , & Bennett, J. R. (2022). Prioritization of public and private land to protect species at risk habitat. Conservation Science and Practice, 4(9), e12771. 10.1111/csp2.12771

[ece39725-bib-0065] Quinby, P. (2020). The Catchacoma ancient Forest landscape: An initial inventory of species and habitats.

[ece39725-bib-0066] Schloss, C. A. , Cameron, D. R. , McRae, B. H. , Theobald, D. M. , & Jones, A. (2022). “No‐regrets” pathways for navigating climate change: Planning for connectivity with land use, topography, and climate. Ecological Applications, 32(1), e02468. 10.1002/eap.2468 34614272 PMC9285781

[ece39725-bib-0067] Schuster, R. , Germain, R. R. , Bennett, J. R. , Reo, N. J. , & Arcese, P. (2019). Vertebrate biodiversity on indigenous‐managed lands in Australia, Brazil, and Canada equals that in protected areas. Environmental Science & Policy, 101, 1–6. 10.1016/j.envsci.2019.07.002

[ece39725-bib-0068] Secretariat of the CBD . (2020). Update of the Zero Draft of the Post‐2020 Global Biodiversity Framework.

[ece39725-bib-0069] Seddon, N. , Chausson, A. , Berry, P. , Girardin, C. A. J. , Smith, A. , & Turner, B. (2020). Understanding the value and limits of nature‐based solutions to climate change and other global challenges. Philosophical Transactions of the Royal Society B, 375(1794), 20190120. 10.1098/rstb.2019.0120 PMC7017763 31983344

[ece39725-bib-0070] Seddon, N. , Smith, A. , Smith, P. , Key, I. , Chausson, A. , Girardin, C. , House, J. , Srivastava, S. , & Turner, B. (2021). Getting the message right on nature‐based solutions to climate change. Global Change Biology, 27(8), 1518–1546. 10.1111/gcb.15513 33522071

[ece39725-bib-0071] Shin, Y. , Midgley, G. F. , Archer, E. R. M. , Arneth, A. , Barnes, D. K. A. , Chan, L. , Hashimoto, S. , Hoegh‐Guldberg, O. , Insarov, G. , Leadley, P. , Levin, L. A. , Ngo, H. T. , Pandit, R. , Pires, A. P. F. , Pörtner, H. , Rogers, A. D. , Scholes, R. J. , Settele, J. , & Smith, P. (2022). Actions to halt biodiversity loss generally benefit the climate. Global Change Biology, 28(9), 2846–2874. 10.1111/gcb.16109 35098619 PMC9303674

[ece39725-bib-0072] Smith, R. J. , Bennun, L. , Brooks, T. M. , Butchart, S. H. M. , Cuttelod, A. , Di Marco, M. , Ferrier, S. , Fishpool, L. D. C. , Joppa, L. , Juffe‐Bignoli, D. , Knight, A. T. , Lamoreux, J. F. , Langhammer, P. , Possingham, H. P. , Rondinini, C. , Visconti, P. , Watson, J. E. M. , Woodley, S. , Boitani, L. , … Scaramuzza, C. A. d. M. (2019). Synergies between the key biodiversity area and systematic conservation planning approaches. Conservation Letters, 12(1), e12625. 10.1111/conl.12625

[ece39725-bib-0073] Smith, W. , & Cheng, R. (2016). Canada's intact forest landscapes updated to 2013. Ottawa.

[ece39725-bib-0074] Sothe, C. , Gonsamo, A. , Arabian, J. , Kurz, W. A. , Finkelstein, S. A. , & Snider, J. (2022). Large soil carbon storage in terrestrial ecosystems of Canada. Global Biogeochemical Cycles, 36(2), e2021GB007213. 10.1029/2021GB007213

[ece39725-bib-0075] Soto‐Navarro, C. , Ravilious, C. , Arnell, A. , De Lamo, X. , Harfoot, M. , Hill, S. L. L. , Wearn, O. R. , Santoro, M. , Bouvet, A. , Mermoz, S. , Le Toan, T. , Xia, J. , Liu, S. , Yuan, W. , Spawn, S. A. , Gibbs, H. K. , Ferrier, S. , Harwood, T. , Alkemade, R. , … Kapos, V. (2020). Mapping co‐benefits for carbon storage and biodiversity to inform conservation policy and action. Philosophical Transactions of the Royal Society B, 375(1794), 20190128. 10.1098/rstb.2019.0128 PMC7017768 31983334

[ece39725-bib-0076] Spencer, W. D. , Beier, P. , Penrod, K. , Winters, K. , Paulman, C. , Rustigian‐Romsos, H. , Strittholt, J. , Parisi, M. , & Pettler, A. (2010). California essential habitat connectivity project: A strategy for conserving a connected California. Prepared for California Department of Transportation, California Department of Fish and Game, and federal highways administration. http://www.scwildlands.org/reports/CaliforniaEssentialHabitatConnectivityProject.pdf

[ece39725-bib-0077] Stralberg, D. , Arseneault, D. , Baltzer, J. L. , Barber, Q. E. , Bayne, E. M. , Boulanger, Y. , Brown, C. D. , Cooke, H. A. , Devito, K. , Edwards, J. , Estevo, C. A. , Flynn, N. , Frelich, L. E. , Hogg, E. H. , Johnston, M. , Logan, T. , Matsuoka, S. M. , Moore, P. , Morelli, T. L. , … Whitman, E. (2020). Climate‐change refugia in boreal North America: What, where, and for how long? Frontiers in Ecology and the Environment, 18(5), 261–270. 10.1002/fee.2188

[ece39725-bib-0078] Stralberg, D. , Carroll, C. , & Nielsen, S. E. (2020). Toward a climate‐informed north American protected areas network: Incorporating climate‐change refugia and corridors in conservation planning. Conservation Letters, 13(4), e12712. 10.1111/conl.12712

[ece39725-bib-0079] Theobald, D. M. , Reed, S. E. , Fields, K. , & Soulé, M. (2012). Connecting natural landscapes using a landscape permeability model to prioritize conservation activities in the United States: Connecting natural landscapes. Conservation Letters, 5(2), 123–133. 10.1111/j.1755-263X.2011.00218.x

[ece39725-bib-0080] Thompson, P. L. , Rayfield, B. , & Gonzalez, A. (2017). Loss of habitat and connectivity erodes species diversity, ecosystem functioning, and stability in metacommunity networks. Ecography, 40(1), 98–108. 10.1111/ecog.02558

[ece39725-bib-0081] Townsend, J. , Moola, F. , & Craig, M. K. (2020). Indigenous peoples are critical to the success of nature‐based solutions to climate change. Facets, 5(1), 551–556. 10.1139/FACETS-2019-0058

[ece39725-bib-0082] Turney, C. , Ausseil, A.‐G. , & Broadhurst, L. (2020). Urgent need for an integrated policy framework for biodiversity loss and climate change. Nature Ecology Evol., 4(8), 996. 10.1038/s41559-020-1242-2 PMC7299142 32555535

[ece39725-bib-0083] USEPA . (2015). Greenhouse gas equivalencies calculator. USEPA.

[ece39725-bib-0084] Valach, A. C. , Kasak, K. , Hemes, K. S. , Anthony, T. L. , Dronova, I. , Taddeo, S. , Silver, W. L. , Szutu, D. , Verfaillie, J. , & Baldocchi, D. D. (2021). Productive wetlands restored for carbon sequestration quickly become net CO2sinks with site‐level factors driving uptake variability. PLoS One, 16(3), e0248398. 10.1371/journal.pone.0248398 33765085 PMC7993764

[ece39725-bib-0085] Wang, C. , Zhang, W. , Li, X. , & Wu, J. (2021). A global meta‐analysis of the impacts of tree plantations on biodiversity. Global Ecology and Biogeography, 31, 576–587. 10.1111/geb.13440

[ece39725-bib-0086] Ward, M. , Saura, S. , Williams, B. , Ramírez‐Delgado, J. P. , Arafeh‐Dalmau, N. , Allan, J. R. , Venter, O. , Dubois, G. , & Watson, J. E. M. (2020). Just ten percent of the global terrestrial protected area network is structurally connected via intact land. Nature Communications, 11(1), 4563. 10.1038/s41467-020-18457-x PMC7486388 32917882

[ece39725-bib-0087] Watson, J. E. M. , Evans, T. , Venter, O. , Williams, B. , Tulloch, A. , Stewart, C. , Thompson, I. , Ray, J. C. , Murray, K. , Salazar, A. , McAlpine, C. , Potapov, P. , Walston, J. , Robinson, J. G. , Painter, M. , Wilkie, D. , Filardi, C. , Laurance, W. F. , Houghton, R. A. , … Lindenmayer, D. (2018). The exceptional value of intact forest ecosystems. Nature Ecology Evolution, 2(4), 599–610. 10.1038/s41559-018-0490-x 29483681

[ece39725-bib-0088] Wells, J. V. , Dawson, N. , Culver, N. , Reid, F. A. , & Morgan Siegers, S. (2020). The state of conservation in North America's boreal Forest: Issues and opportunities. Front. For. Glob. Change, 3, 90. 10.3389/ffgc.2020.00090

[ece39725-bib-0089] Zhu, L. , Hughes, A. C. , Zhao, X. Q. , Zhou, L. J. , Ma, K. P. , Shen, X. L. , Li, S. , Liu, M. Z. , Xu, W. B. , & Watson, J. E. M. (2021). Regional scalable priorities for national biodiversity and carbon conservation planning in Asia. Science Advances, 7(35), eabe4261. 10.1126/sciadv.abe4261 34446433 PMC8388611

