# Supplementary material for: Integrating carbon stocks and landscape connectivity for nature‐based climate solutions
Source: Ecol Evol. 2023 Jan 9;13(1):e9725. doi: 10.1002/ece3.9725 (PMC9829451; doi:10.1002/ece3.9725)
Supplement: Supplementary file 1 — Appendix S1: [file ECE3-13-e9725-s001.docx]

**Appendix A1**


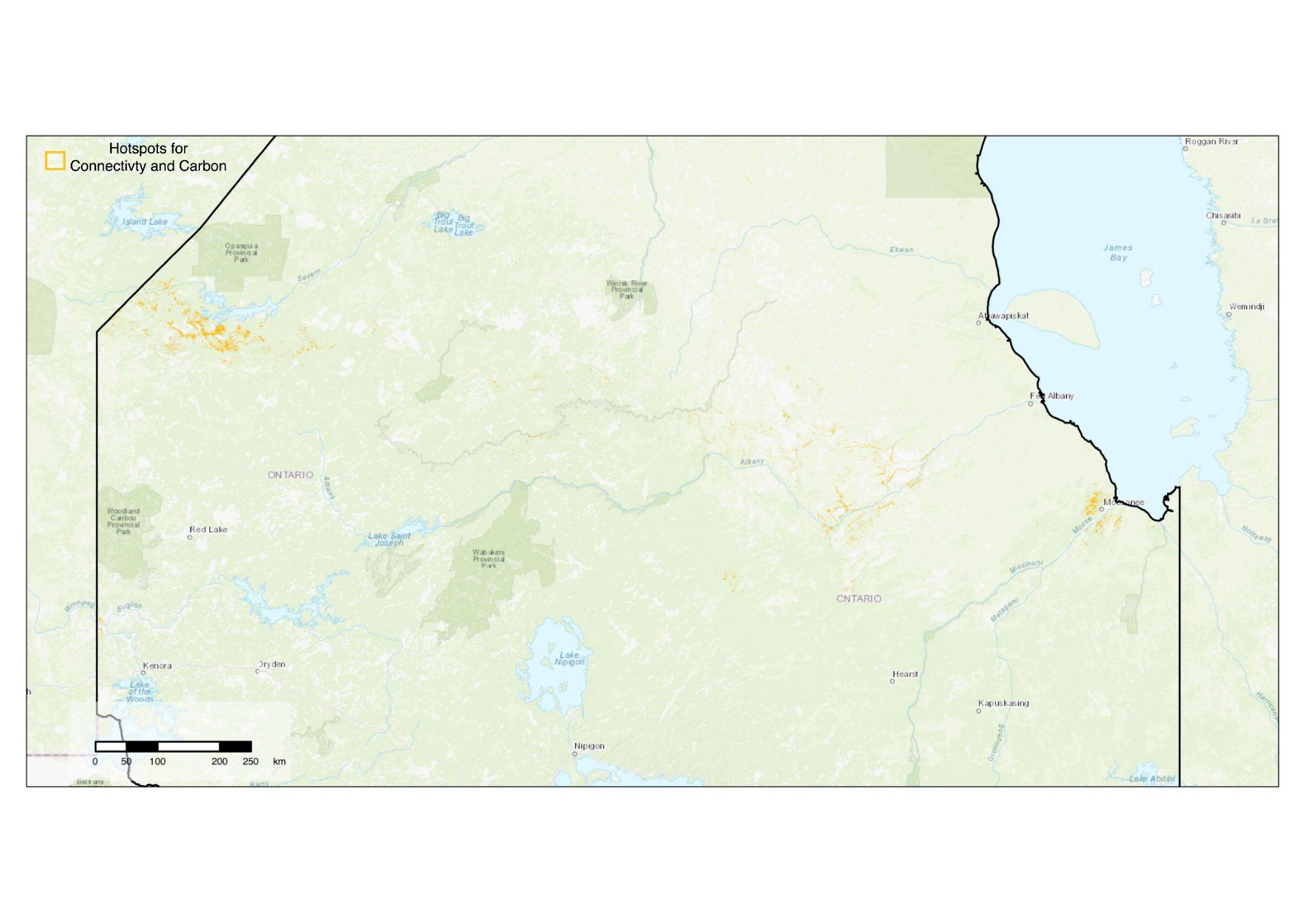


**Fig. A1.** Hotspots for landscape connectivity, forest carbon, and soil carbon in Ontario, Canada. Yellow regions represent 300x300m raster cells that are in the top 20% quantile for current density, forest carbon and soil carbon.
